# Retinal injuries in seven teenage boys from the same handheld laser

**DOI:** 10.1016/j.ajoc.2022.101596

**Published:** 2022-05-25

**Authors:** Sayed Faraj, Marianne Etzelmüller Bathen, Augustinas Galeckas, Andreas Myrold, Ingar Stene-Johansen, Øystein Kalsnes Jørstad, Morten Carstens Moe

**Affiliations:** a Department of Ophthalmology, Oslo University Hospital, Norway; b Institute for Clinical Medicine, Faculty of Medicine, University of Oslo, Norway; c Advanced Power Semiconductor Laboratory, ETH Zürich, Switzerland; d Department of Physics/ Centre for Materials Science and Nanotechnology, Faculty of Mathematics and Natural Sciences, University of Oslo, Norway

**Keywords:** Laser-induced maculopathy, Laser-induced retinal injury, Laser-induced retinopathy, Choroidal neovascularization

## Abstract

This paper presents retinal injuries in 10 eyes of seven teenagers who had been playing with a handheld laser. They reported different degrees of visual symptoms in the form of central scotomas. Clinical examination revealed light burns in the maculae and disruption of the retinal layers on OCT. One patient developed secondary choroidal neovascularization (CNV), which was successfully treated with intravitreal ranibizumab. For some of the patients, the injuries led to permanent visual sequela. This devastating case series emphasizes the need for awareness among minors, parents and communities about the danger of playing with handheld lasers.

## 1 Introduction

Exposure to lasers light can result in permanent vision loss. Minors are at particular risk for careless use of handheld lasers, and the easy availability of even strong lasers is a public health concern. An increasing number of retinal injuries from laser handhelds have been reported since the late 1990s; a systematic review from 2017 found 48 publications describing a total of 111 patients (137 eyes) with such injuries.^1^

The International Electrochemical Commission (IEC) (IEC 60825–1) classifies lasers from 1 to 4 in correspondence to the risk they represent.^2^ The American National Standards Institute (ANSI) uses the same classification. Laser pointers are classified as 1, 2 or 3R lasers. Handheld lasers, on the other hand, are classified as 3B or 4, and their use is regulated by law in many countries. Still, laser pointers and handheld lasers can be indistinguishable by appearance, and the labeling may even be erroneous.^3^

There are reports of laser shone at pilots, posing a potential risk of serious accidents.^4^ A laser beam can cause thermal, photochemical and/or mechanical injury to the eye.^5^ The damage potential depends on the power output and wavelength of the laser and the exposure time. Since the eye focuses light onto the central retina, the macula is particularly vulnerable. Accordingly, laser injuries typically impair the central vision. In this devastating case series, seven teenagers who played with a high-powered handheld laser suffered retinal injuries.

## 2 Findings

**Fig. 1:**
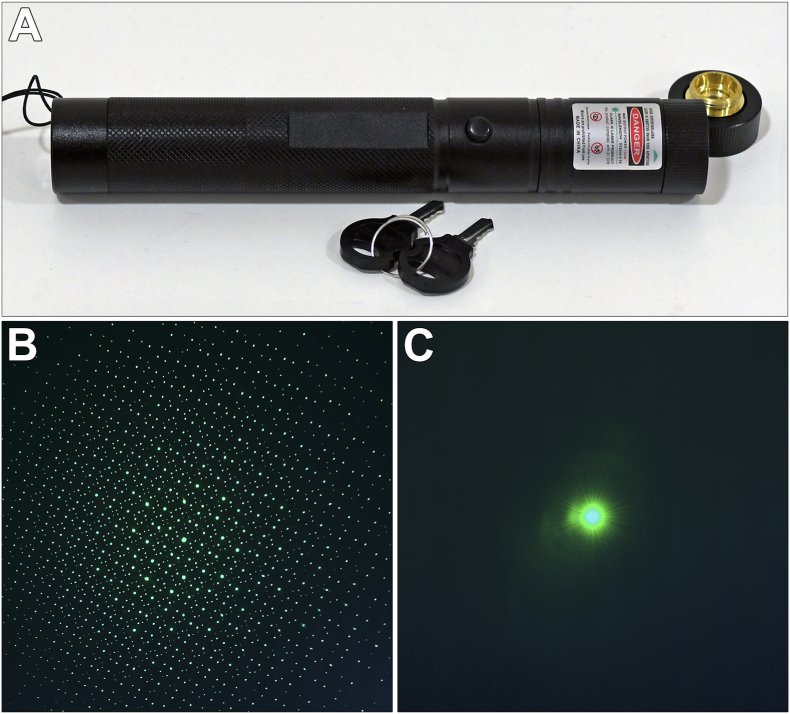
Photograph of the handheld laser. The laser could be turned on or off with a safety key. It was labeled class III laser product and marked with a laser warning sign (**A**). By default, it had a diffraction grating lens attached (**B**). Without the diffraction lens, however, the laser presented a single green laser beam with approximately 16 times the labeled output power (**C**). (For interpretation of the references to color in this figure legend, the reader is referred to the Web version of this article.)

In January 2020, a group of teenage boys who had been playing with a handheld laser purchased online were referred to the Department of Ophthalmology at Oslo University Hospital because of concerns about visual symptoms. The handheld laser was labeled “Max output power <5 mW - Wavelength 532±10 nm - Class III Laser Product” (Fig. 1A). It was also marked with a laser warning sign. By default, the handheld laser had a diffraction grating lens attached, but this had been easily removed by the teenagers (Fig. 1B). Because of the severe clinical presentation, we suspected that the laser was stronger than labeled. Its output power was therefore measured at the Department of Physics at the University of Oslo. Without the diffractive lens attached (Fig. 1C), the laser created a single beam of 80–90 mW, i.e., at least 16 times the labeled output power. The wavelength was measured to 532 nm (green color), which was according to label.

The patients were examined at presentation (baseline) and regularly followed for 12 months. Best-corrected visual acuity (BCVA) was obtained with a ClearChart (Reichert Technologies, Depew, NY) digital acuity test, which displays five letter optotypes per line and logarithm of the minimal angle of resolution line size progression. Retinal images were obtained with ultra-widefield scanning laser ophthalmoscopy (Optomap P200Tx, Optos, Dunfermline, the UK), spectral-domain optical coherence tomography (OCT) (RS-3000 Advance, NIDEK CO., LTD., Gamagori, Japan), and swept-source OCT angiography (OCT-A) (PLEX Elite 9000, ZEISS, Oberkochen, Germany).

Seven patients had evidence of macular injury. In three of these seven patients, both eyes were affected. Four of seven patients reported the exposure to be self-inflicted, whereas three told that the exposure was inflicted on them by one of the others. None of them had any relevant pre-existing medical or ophthalmic conditions. In the following we present a summary of the cases.

### 2.1 Case 1

**Fig. 2:**
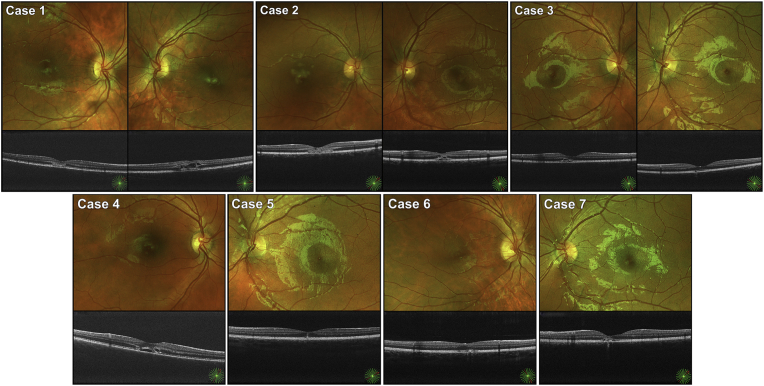
Fundus photographs and cross-sectional optical coherence tomography (OCT) scans of Case 1–7 at presentation. Case 1–3 had bilateral macular injuries, whereas case 4–7 only had injuries in one eye. The photographs and OCT scans show varying degrees of pale spots in the central macula and disruption of the other retinal layers.

**Fig. 3:**
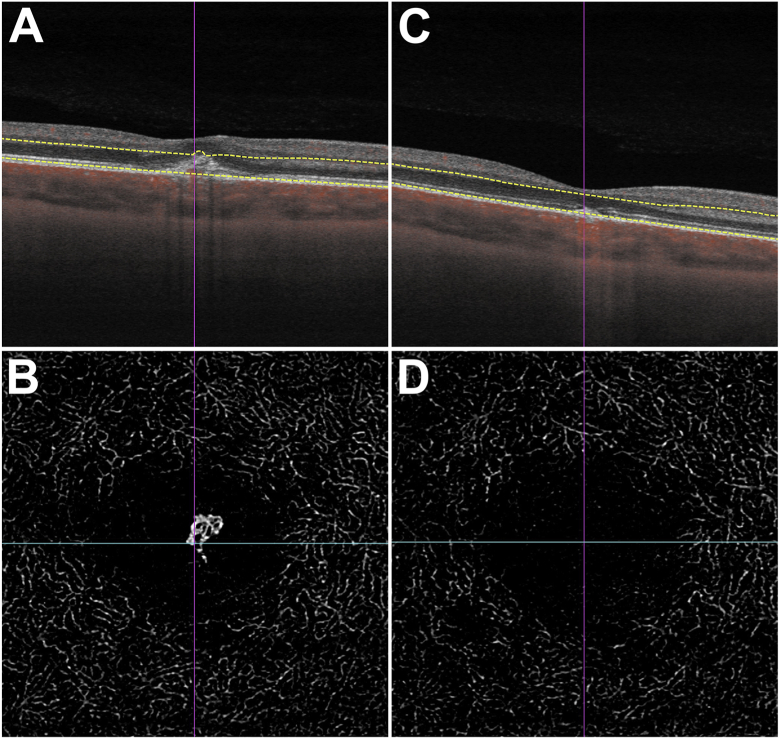
Optical coherence tomography (OCT) of the right eye of Case 1. Cross-sectional macula scan (**A**) shows central retinal thickening and a hyperreflective lesion in the subretinal space. OCT angiography (OCT-A) reveals vascular branching in the outer retina (**B**). These findings are consistent with a type 2 choroidal neovascularization. After four monthly intravitreal injections with ranibizumab, there is resolution of retinal thickening on OCT (**C**) and choroidal neovascularization on OCT-A (**D**).

At presentation this patient complained about headache and central scotomas, the latter was more prominent in the left eye. BCVA was 20/32 in the right eye and 20/40 in the left eye. Funduscopic examination revealed multiple pale spots in both maculae (Fig. 2). After initial improvement the patient experienced sudden worsening of vision in the right eye three weeks after the injury. OCT and OCT-A revealed a type 2 choroidal neovascularization (CNV) in the right fovea next to a laser burn (Fig. 3A/B).

The CNV in the right eye was treated with intravitreal ranibizumab in accordance with a pro re nata (PRN) regimen. Four monthly ranibizumab injections resulted in visual improvement and sustained remission of the CNV (Fig. 3C/D). After six weeks, OCT also gave suspicion of CNV in the left macula, which was initially treated with one aflibercept injection and then two monthly ranibizumab injections (the choice of drug was at the discretion of the treating physician). However, there was no functional or anatomical improvement. After reevaluation the structural changes in the left macula these were regarded as atrophic, and no further therapy was given. At the final visit after one year, the patient reported slight metamorphopsia in the left eye, but the central scotomas had recovered. BCVA was 20/25 in the right eye and 20/20 in the left eye. There was hyperpigmentation and some atrophy in both maculae. Correspondingly, OCT displayed hyporeflective changes in the outer retinal layers.

### 2.2 Case 2

At presentation this patient complained about central scotomas in both eyes. BCVA was 20/40 in the right eye and 20/32 in the left eye. Funduscopic examination revealed multiple pale spots in both maculae (Fig. 2). On OCT there were hyperreflective changes centrally in the right macula. At follow-up after three weeks, a hyperreflective subretinal lesion had developed. A watchful waiting strategy was pursued without CNV developing. At the final visit after one year, the patient's experience of central scotomas had improved. BCVA was 20/25 in both eyes. There was parafoveal hyperpigmentation in both eyes. Correspondingly, OCT displayed hyporeflective changes in the outer layers of the left macula.

### 2.3 Case 3

At presentation this patient complained about central scotomas bilaterally. BCVA was 20/16 in both eyes. Funduscopic examination revealed a single pale spot in both maculae (Fig. 2). On OCT of the right macula, there was disruption of the outer retinal layers with slight subretinal edema. During the next weeks, the patient developed headache, binocular visual disturbances and increasing central scotomas. BCVA decreased to 20/50 in both eyes. By contrast, there was gradual resolution of the OCT abnormalities, and no treatment was given. At the final visit after one year, the patient still experienced small central scotomas bilaterally. BCVA had gradually improved to 20/25 in both eyes. There was small area of atrophy in both maculae. Correspondingly, OCT displayed small defects in the outer retinal layers.

### 2.4 Case 4-7

These four patients each had clinical findings consistent with mild laser-induced retinal injury in one eye. Table 1 presents a summary of Case 1–7.

**Table 1:** A summary of Case 1–7. BCVA: Best-corrected visual acuity.

Case	Eye	Initial BCVA	Symptomatic scotoma at presentation	BCVA after 12 months
				
1	Both	20/32–20/40	Yes	20/25–20/20
2	Both	20/40–20/32	Yes	20/25–20/25
3	Both	20/16–20/16	Yes	20/25–20/25
4	Right	20/25	Yes	20/16
5	Left	20/20	No	20/16
6	Right	20/16	No	20/16
7	Left	20/20	Yes	20/12

## 3 Discussion

This case series describes retinal injuries in seven teenage boys from the same high-powered handheld laser. The severity of the injuries differed, and in one case secondary CNV developed. Three patients suffered visual sequela.

The funduscopic appearance and retinal disruption on OCT were typical for laser-induced retinal injures: a yellowish lesion corresponding to the thermal injury in the acute phase and transition to hyper- and hypopigmented alterations in the chronic phase.^6^, ^7^ Also, laser-induced retinal injuries have been reported to lead secondary CNV in a handful of previous cases reports^8^, ^9^, ^10^; and a few of these have been treated with anti-VEGF. A 14-year-old Danish patient, for instance, achieved good long term effect of two intravitreal ranibizumab injections.^11^ Similarly, four monthly ranibizumab injections resulted in sustained CNV remission in our patient (case 1). Taken together, this demonstrates that PRN can be an appropriate treatment regimen for laser-induced CNV, and that prolonged anti-VEGF treatment may not be necessary.

In general, the output power of a laser cannot be higher than 1 mW (Class 1 or 2) to be considered safe under normal conditions.^2^ Still, the energy (joules) that a laser releases is the product of its output power (watts) and duration (seconds). Accordingly, even a weak laser can cause retinal damage after prolonged exposure. The retinal injuries in this case series were caused by a laser with an output power of 80–90 mW and wavelength of 532 nm, which corresponding to a relatively powerful Class 3B laser (5–500 mW; > 315 nm). Needless to say, Class 3B lasers are relatively strong and harbour a high risk of eye injuries. Another striking feature of this case series is that minors had obtained such a strong handheld laser. Online shopping is of particular concern in this regard. Entering keywords such as “buy strong laser” in any popular search engine results in numerous options for acquiring high-powered lasers. While the Norwegian Radiation and Nuclear Safety Authority, for instance, has proposed a European ban on powerful laser pointers,^12^ Internet shopping is in reality notoriously difficult to regulate. Sad and unfortunate as this case series is, it is a price we pay for a global online marketplace. It nonetheless underscores the need for building public recognition of laser safety and also parental awareness and monitoring of adolescent Internet use.

The prognosis of laser-induced retinal injury has been reviewed by Birtel et al.; more than half of the patients presented with BCVA less than 20/40, but78% had an increase in BCVA after six months. The visual prognosis depends on the extent and location of the retinal damage. Exposure to high-powered lasers may even require surgical intervention.^13^, ^14^ All patients in this case series presented with a BCVA equal to or better than 20/40 at presentation, and fortunately all achieved a final BCVA of at least 20/25. Still, three patients reported persisting scotomas, but no disability in activities of daily living.

In summary, handheld lasers pose a risk of retinal injuries. In this case series such injuries were found in 10 eyes of seven teenagers who had been playing with a strong handheld laser. This devastating event emphasizes the need for awareness among minors, parents and communities about the danger of handheld lasers.

## 4 Patient consent

Written informed consent was obtained from all patients, and publication was approved by the Institutional data protection officer at Oslo University Hospital.

## Financial support

None.

## Declaration of competing interest

ØKJ has been member of Bayer, Allergan and Roche advisory boards and has received lecture fees from Bayer and Allergan. MCM has been member of Bayer, Novartis, Roche and Allergan advisory boards and has received lecture fees from 10.13039/100004326Bayer and 10.13039/100004337Roche. None of the other authors have any relevant conflicts of interest.

The authors would like to thank Geir A. Qvale for preparing the figures.
